# New findings on choroidal features in healthy people by ultra-widefield swept-source optical coherence tomography angiography

**DOI:** 10.1038/s41598-023-36374-z

**Published:** 2023-06-06

**Authors:** Xinyue Liu, Sizhu Chen, Hongmei Leng, Yiya Wang, Yi Liu, Yadan Shen, Sanmei Liu, Hangjin Yi, Jie Li, Jie Zhong

**Affiliations:** 1 School of Medicine, University of Electronic Science and Technology of China, No.23, West Section 2, 1St Ring Road, Qing Yang District, Chengdu City, 610072 Sichuan Province China; 2 Department of Ophthalmology, Sichuan Provincial People’s Hospital, School of Medicine, University of Electronic Science and Technology of China, Chengdu, 610072 Sichuan China; 3 Chengdu University of Traditional Chinese Medicine, Chengdu, China

**Keywords:** Biotechnology, Computational biology and bioinformatics, Physiology, Structural biology, Anatomy, Health care, Health occupations, Medical research, Pathogenesis, Risk factors, Engineering, Physics

## Abstract

To evaluate the distribution of choroidal thickness (CT) and its trend with age in healthy people using 120° ultra-wide field swept-source optical coherence tomography angiography (UWF SS-OCTA). In this cross-sectional observational study, healthy volunteers underwent single imaging of the fundus with UWF SS-OCTA at a field of view (FOV) of 120° (24 mm × 20 mm) centered on the macula. The characteristics of CT distribution in different regions and its changes with age were analyzed. A total of 128 volunteers with a mean age of 34.9 ± 20.1 years and 210 eyes were enrolled in the study. The thickest mean choroid thickness (MCT) was located at the macular region and supratemporal region, followed by the nasal side of the optic disc, and thinnest below the optic disc. The maximum MCT was: 213.40 ± 36.65 μm for the group aged 20–29, and the minimum MCT was: 162.11 ± 31.96 μm for the group aged ≥ 60. After the age of 50, MCT was significantly and negatively correlated decreased with age (r = − 0.358, *p* = 0.002), and the MCT in the macular region decreased more remarkably compared to other regions. The 120° UWF SS-OCTA can observe the distribution of choroidal thickness in the range of 24 mm × 20 mm and its variation with age. It was revealed that MCT decreased more rapidly in the macular region relative to other regions after 50 years old.

## Introduction

The choroid is a layer of blood vessel-rich tissue located between the retina and the sclera. It has the functions of supplying nutrients and oxygen needed by the retina, changing the position of the retina through choroid thickness (CT), regulating body temperature, and secreting various growth factors^1^. Obtaining choroidal thickness and other parameters in healthy people is of great significance for the diagnosis of diseases related to choroidal changes, such as high myopia, diabetic retinopathy, thick choroidal disease, and age-related macular degeneration (AMD)^2–5^.

Previous methods such as histological studies and A-scan ultrasonography measurements have been employed to measure CT^6,7^, while they were inadequate owing to the higher accuracy required for in vivo CT measurements^8^.

Optical coherence tomography (OCT) and optical coherence tomography angiography (OCTA) can provide information about the retinal, choroidal structure, and blood flow^9–11^, with the advantages of non-invasiveness, high resolution, rapid operation, and good reproducibility^12–15^.

Numerous researchers have employed spectral-domain optical coherence tomography(SD-OCT) and swept-source optical coherence tomography(SS-OCT) to measure CT. Nonetheless, they were limited by the scope of measurement, the age of participants, and the sample size. Touhami S, et al. (2020) analyzed 12 mm x 12 mm CT topography using a wide range of SS-OCTA and discovered an asymmetric distribution of choroidal thickness over a larger area^16^.

The latest ultra-wide field (UWF) SS-OCTA device (BM-400 K BMizar, Toward Pi Medical Technologies, Beijing, China), which has 400 kHz sweep source technology capable of acquiring fundus blood flow maps of 24 mm × 20 mm in a single within 15 s, provides access to the mean choroid thickness (MCT) and vessel density (VD) by region through its quantification software. It allows the study of the choroidal structure at a larger scale and in a more visual way. To our knowledge, this is the largest range of studies evaluating choroidal features among healthy individuals.

For the first time, the latest high-speed UWF SS-OCTA was used to obtain images in the 120° range of the posterior pole to analyze the CT distribution in different regions of the fundus and its characteristics with the age of healthy people.

## Material and methods

### Subjects

This was a cross-sectional observational study involving 128 healthy subjects who underwent eye examinations at Sichuan Provincial People's Hospital between November 2021 and May 2022. All participants signed the informed consent before the examination. This study was approved by the Ethics Committee of Sichuan Provincial People's Hospital. All procedures adhere to the principles outlined in the Declaration of Helsinki.

The inclusion criteria were (1) Intraocular Pressure (IOP) ≤ 21 mmHg in both eyes; (2) 26 mm ≥ Axis Length (AL) ≥ 22 mm; (3) + 3.00D ≥ Refractive Error (RE) ≥  − 6.00D; (4) Best Corrected Visual Acuity (BCVA, expressed using the decimal recording method) ≥ 0.8; (5) Imaging quality score ≥ 7. Exclusion criteria were (1) ocular diseases; (2) a history of ocular trauma and surgery; (3) systemic chronic diseases, such as diabetes, hypertension, and hyperthyroidism; (4) ocular media opacity or motion artifacts that prevent high-quality imaging or layering artifacts that cannot be corrected manually.

### Ophthalmologic examination

Recruited volunteers underwent a comprehensive eye examination consisting of refraction and BCVA, IOP, AL, slit lamp examination, color fundus photography, and OCT/OCTA. The subjects were examined during the time period between 10–12 am and 2–5 pm while resting in a quiet state for 15 min prior to the examination.

### Image acquisition protocol

**Figure 1:**
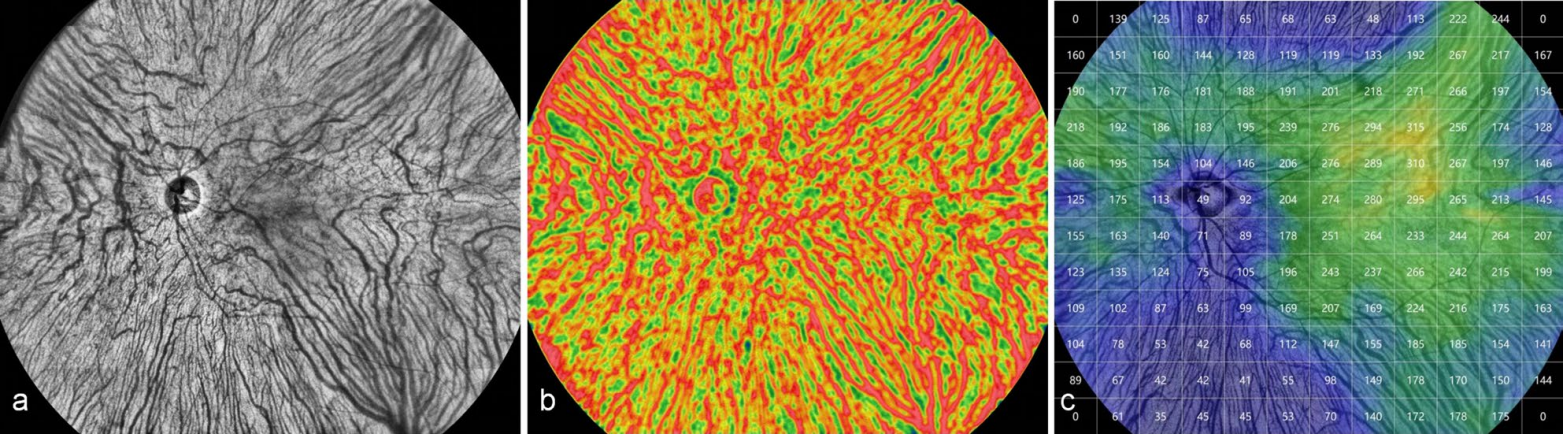
Representative 24 mm × 20 mm images of a 56-year-old healthy female (left eye) obtained by the UWF SS-OCTA. a. En face image of large vessels in the choroid; b. blood flow density map of large vessels in the choroid; c. quantified topography of choroidal thickness.

The OCTA allows for blood flow imaging in a single scan centered on the macula in a 24 mm × 20 mm area with a total field of view (FOV) of 120°. It adopts a vertical cavity laser with a wavelength of 1060 nm and a scanning speed of 400,000 A-scans/second, providing a lateral resolution of 10 μm and an axial resolution of 3.8 μm (optical). The instrument has an A-scan depth of 6.0 mm (2560 pixels) in the tissue. The scanning range of 24 mm × 20 mm consists of 1536 A-scans and 1280 B-scans. The interval between each A-scan and B-scan is 15.625 μm. It performs two consecutive B-scans at each fixation position and then proceeds to the next lateral position on the retina for scanning (Fig. 1a).

The data collection was performed by the same trained examiner. CT was defined as the distance between Bruch's membrane (the lower border of the retinal pigment epithelium (RPE) and the choroid-sclera interface. Automatic stratification and measurements and provided by the built-in software. Manual correction of retinal segmentation was performed only when automatic stratification was inaccurate (Fig. 1b and c).

### Data analysis

Statistical analyses were conducted by IBM SPSS Statistics 26.0 (IBM Corporation, New York, USA). Continuous variables were expressed as mean ± standard deviation. Shapiro–wilk test was employed to examine the normality of the data. Regarding normally distributed data, continuous variables were analyzed using independent sample t-tests between two groups, and differences between multiple groups were investigated using one-way ANOVA with LSD Test. Then, nonparametric tests were utilized to analyze non-normally distributed data. Differences in continuous variables between two groups were explored using the Mann–Whitney U test, and differences in continuous variables between multiple groups were analyzed using the Kruskal–Wallis test. The relationship between age and choroidal thickness was researched using Pearson's correlation test. A nominal two-side p value of 0.05 was considered to indicate statistical significance.

### Ethics statement

The studies involving human participants were reviewed and approved by the ethical review committee of Sichuan Provincial People’s Hospital (SPPH). The patients/participants provided their written informed consent to participate in this study.

## Results

### Characteristics and relevant ocular parameters

A total of 159 individuals were recruited and examined for this study, and 31 subjects were excluded for the following reasons: poor image quality (n = 20), best corrected visual acuity less than 0.8 (n = 2), incomplete or missing information (n = 5), glaucoma (n = 2), and the presence of macular or vitreoretinal disease (n = 2). There were 128 subjects with 210 eyes, 111 in the right eyes, and 99 in the left eyes, which finally met the inclusion criteria. The characteristics and ocular parameters of the subjects were listed in Table 1.

**Table 1:** Basic characteristics of the population.

Characteristic	Mean ± SD (Range)
Age	34.9 ± 20.1 (6–79)
Gender(female)	75.2%
AL (mm)	23.7 ± 0.8 (22.25–25.57)
BCVA	0.99 ± 0.1 (0.8–1.0)
MCT (um)	192.8 ± 38.4 (105.9–286.0)
CVI (%)	38.6 ± 4.4 (24.2–50.5)
CSI (%)	61.56 ± 4.7 (49.5–78.6)

Data were presented as mean ± SD or %

AL, Axis Length; BCVA, Best Corrected Visual Acuity; MCT, mean choroid thickness; CVI indicates the ratio of choroidal vascular volume to choroidal volume in the specified area, reflecting the degree of vascular density; CSI indicates the ratio of the choroidal stromal volume to the choroidal volume in the specified area, reflecting the degree of stromal density.

### Distribution of CT

**Figure 2:**
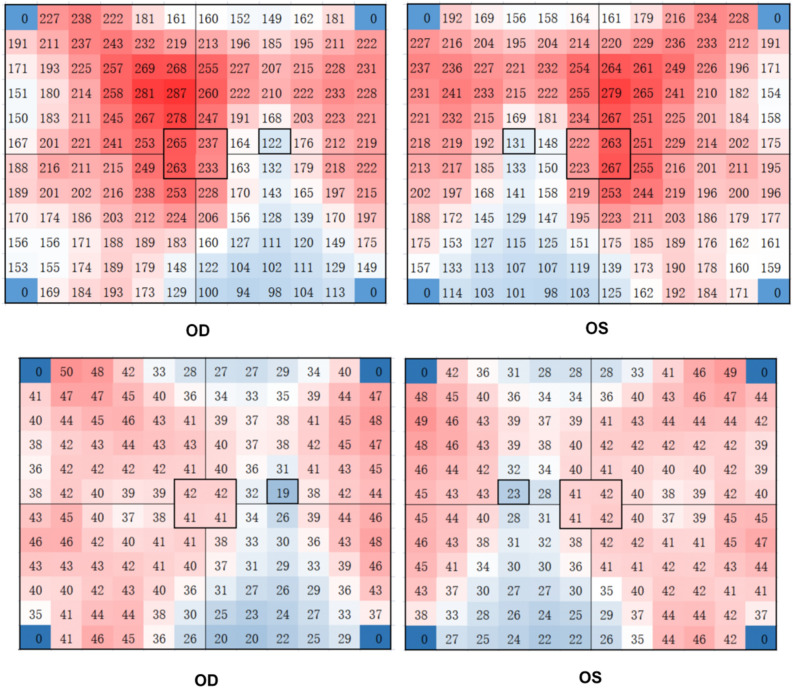
Topography of choroidal grid thickness (top) and topography of density of medium and large vascular layer (Sattler’s layer and Haller’s layer) in the choroid (bottom) for all participants. The darker the red, the thicker the CT in this area; the darker the blue, the thinner the CT in this area. The 4 mm × 3.3 mm area around the fovea was the four small squares surrounded by a black square in the middle, and the center of the macula was in the middle of the four small squares. The optic disc located at 4 mm from the nasal side of the fovea in a 2 mm × 1.7 mm area. Two vertical lines through the macular area divided the other areas into four parts: the supratemporal region, the supranasal region, the inferotemporal region, and the inferonasal region. All subjects followed this division method. This image was generated by Microsoft Excel in version 2021(https://hilcodigital.com/excel/) .

Using a 12 × 12 grid, the 24 mm × 20 mm area was subdivided into 144 small cells with an area of 2 mm × 1.7 mm for clearly exhibiting the distribution of CT in different areas. The quantification software automatically provided the MCT of each small grid. The data for each compartment was averaged and calculated for all subjects to regain the data for each compartment. Accordingly, a topographic map of the mean choroidal grating thickness for all participants was plotted (Fig. 2 top). Similarly, a topographic map of the density of medium and large vascular layer grids in the choroid was depicted (Fig. 2 bottom).

CT of the macular region was measured and compared with its subfoveal choroidal thickness (SFCT). The difference was no statistically significant (*p* = 0.182). The MCT topographic map of all subjects was presented in Fig. 2 (top). The choroid was thickest in the macular and supratemporal regions, thicker on the nasal side of the optic disc, and thinnest on and below the optic disc. The distribution trend of the CVI (%) topographic map was generally consistent with that of CT (Fig. 2 below). Pearson correlation results suggested that MCT was positively correlated with CVI (r = 0.311, *p* < 0.01).

**Figure 3:**
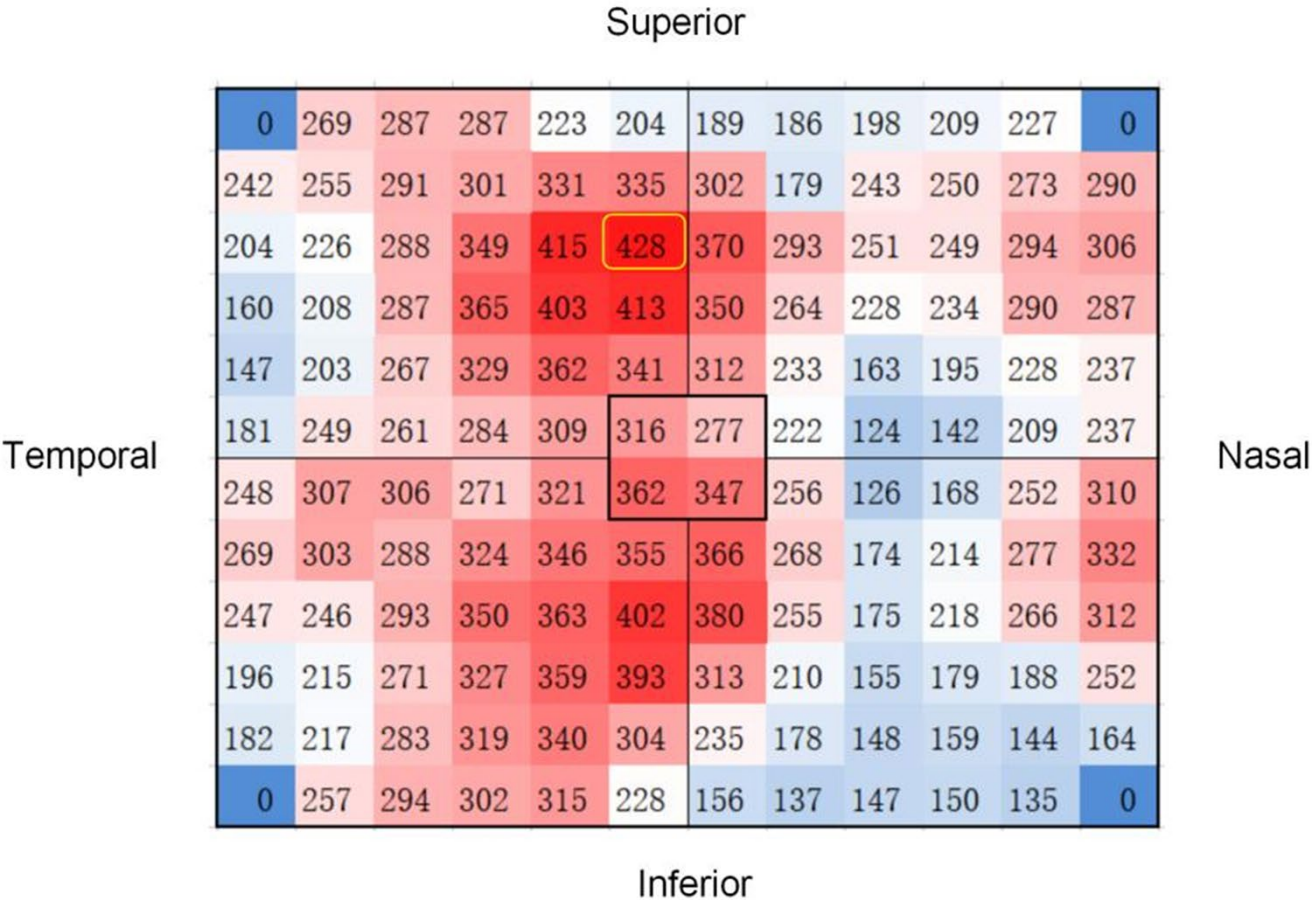
Topographic map of choroidal thickness in the right eye of a 24-year-old healthy man. The small square with the maximum mean thickness was located 6–8 mm above the temporal aspect of the fovea (yellow square). This image was generated by Microsoft Excel in version 2021(https://hilcodigital.com/excel/) .

**Figure 4:**
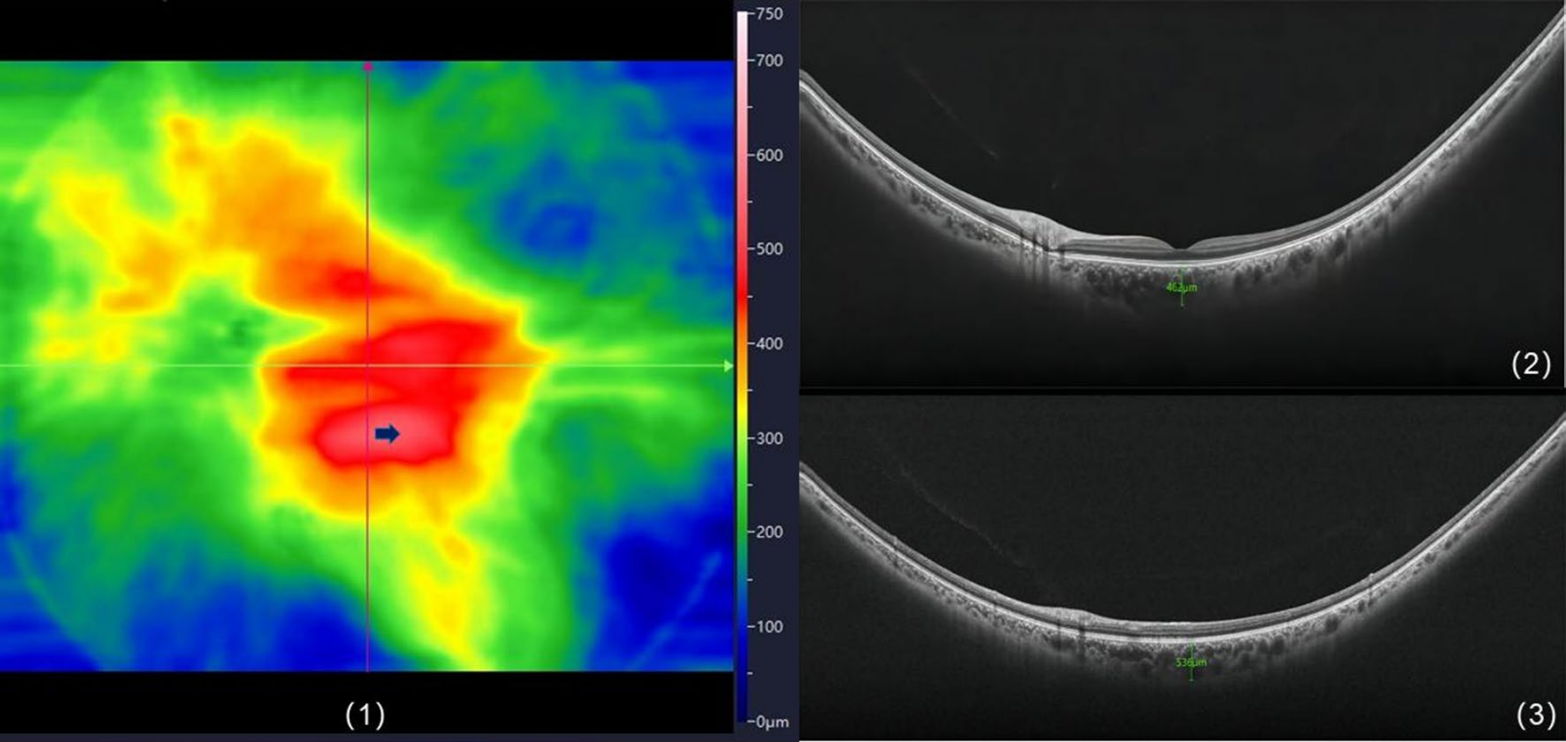
Topographic map of choroidal thickness in the right eye of a 35-year-old woman. (1) B-Scan images of the fovea and the thickest point of the choroid; (2) the SFCT was 462 μm; (3) the TCP (arrow) was 536 μm. TCP, thickest choroidal point. This image was generated by PiView Image System V1^37,38^ (this software is an application built into the device).

We evaluated at the distribution of the squares with maximum values for all subjects. (Fig. 3). The results showed that there were 12 eyes (5.7%) in the macular area, 87 eyes (41.4%) in the supratemporal region, 16 eyes in the inferotemporal region (7.6%), 77 eyes (36.7%) in the supranasal region, and 18 eyes (8.6%) in the inferonasal region. The thickest choroidal point (TCP) was located above the fovea in more than three-quarters of people subfoveal choroidal thickness of eleven eyes (5.2%) was more than 395 μm, and these subjects were aged 20–50 years. B-scan did not reveal pathological dilatation of the choroidal Haller layer, thinning of the Sattler layer and capillary layer, retinal pigment epithelium (REP), and outer retinal lesions (Fig. 4).

### Changes in MCT with age

Next, 210 eyes were divided into six groups following age to further explore the relationship between age and CT: the group aged < 20 (55 eyes), the group aged 20–29 (57 eyes), the group aged 30–39 (17 eyes), the group aged 40–49 (13 eyes), the group aged 50–59 (41 eyes), and the group aged ≥ 60 (27 eyes). The MCT results in different regions for different age groups were shown in Table 2.

**Table 2:** MCT in different regions of different age groups.

Age group(y)	n	Macular	Supratemporal	Inferotemporal	Supranasal	Inferonasal	The whole area
< 20	55	252.61	211.97	184.62	194.57	148.18	188.83
20–29	57	282.59	239.21	200.52	231.72	168.03	213.40
30–39	17	240.40	233.93	183.50	217.41	161.36	199.45
40–49	13	248.15	240.19	184.10	217.43	152.24	199.92
50–59	41	228.36	213.47	176.79	198.24	150.04	184.82
≥ 60	27	192.14	194.54	154.35	166	119.07	162.11

Data were presented as mean.

The whole area means the range of 24 mm × 20 mm.

**Figure 5:**
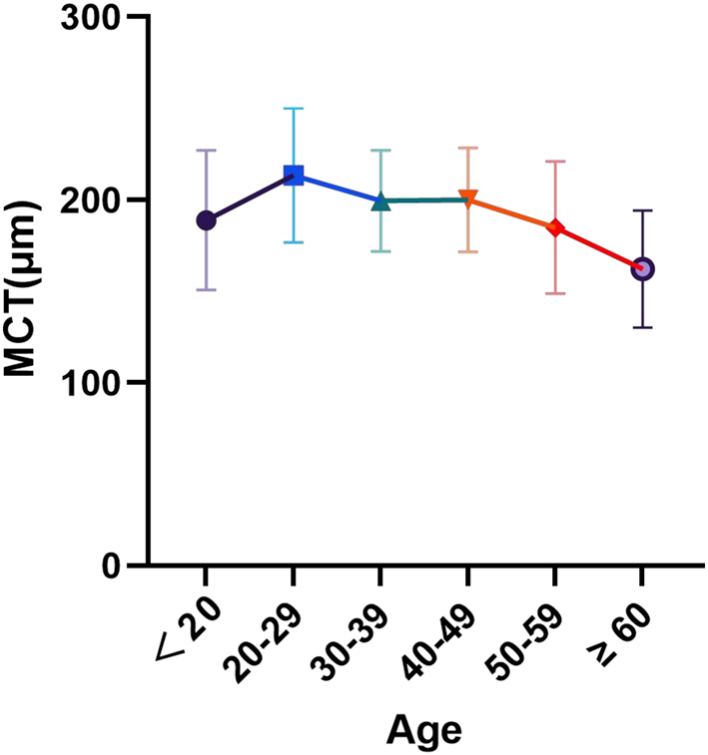
MCT, mean choroid thickness. The whole area means the range of 24 mm × 20 mm.

A line graph of the change in MCT of 24 mm × 20 mm with age was illustrated in Fig. 5. MCT increased and then decreased with age, with the lowest MCT in subjects aged ≥ 60.MCT started to show a decreasing trend over the age of 30. In the group aged 30–49, the Pearson correlation results unveiled no correlation between MCT and age (r = 0.184, *p* = 0.331). However, in the group aged ≥ 50, the whole region (r = − 0.358, *p* = 0.002) exhibited the results negatively correlated with age. Thus, 50 years of age may be an essential time point for the change in MCT with age.

**Figure 6:**
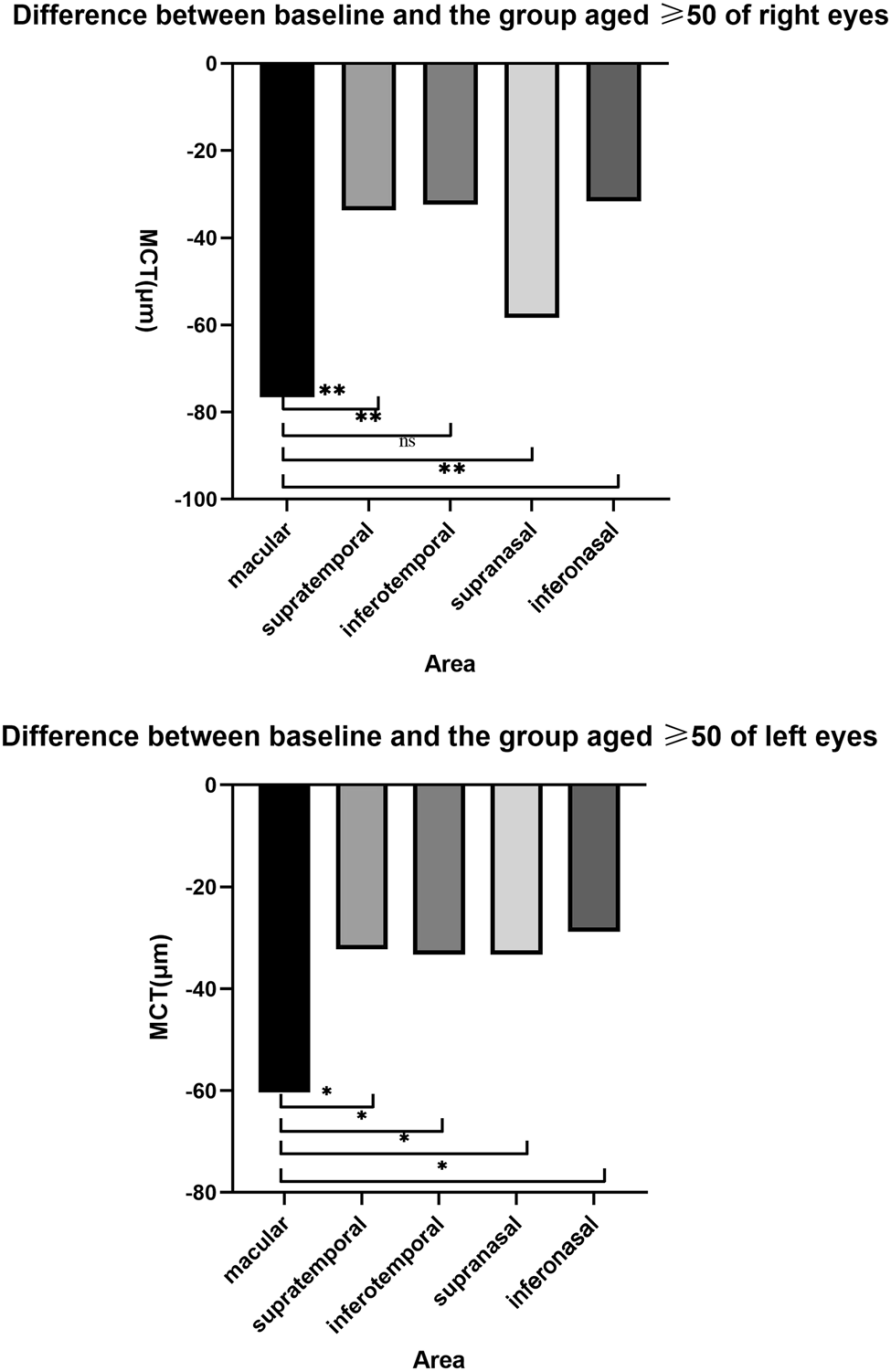
Differences in MCT(μm) between baseline and the group age ≥ 50 of right eyes and left eyes. **p <  0.001 ;*p <  0.05; ns: no significance. MCT, mean choroid thickness.

With the purpose of comparing the variation of MCT in each district after the age of 50, the MCT of the group aged 20–29 was adopted as the baseline, and the difference between the MCT in each region of the group aged ≥ 50 and the baseline was calculated. We separated the left and right eyes to calculate to exclude interocular asymmetry. The One-way ANOVA test and LSD test results revealed that the decrease rate in the macular region of the left eye was greater than that in the supratemporal region (*p* = 0.019), the inferotemporal region (*p* = 0.024), the supranasal region (*p* = 0.024), and the inferonasal region (*p* = 0.009). Although the decrease rate in the macular area of the right eye was not different from the supranasal area (*p* = 0.094), all the other regions were statistically different (*p* <  0.001) (Fig. 6).

## Discussion

The UWF SS-OCTA device has the advantages of ultra-high resolution, longer wavelength (1060 nm), wide range (24 mm × 20 mm, FOV120° range), enhanced penetration, and fast scanning rate (400 kHz)^17^. Our study is the first time to observe the distribution characteristics of choroidal thickness in a 24 mm × 20 mm area using TowardPi UWF SS-OCTA. The choroidal topographic map can directly reflect the distribution of CT in each area and can be followed up longitudinally to observe its changes as an essential follow-up method.

First, previous studies have suggested that the choroid was thickest in the macular region and supratemporal region^18^, with a peak thickness of 5–6 mm on the nasal side of the optic disc, and was thinnest below the optic disc^14^. However, these studies were based on a relatively small imaging range^13,14,19–21^. In our study, these results can be observed in a large area of 24 mm × 20 mm in a single image (Fig. 2, top), while the area of choroidal thickening in the posterior segment is a large semicircular ring around the temporal, superior, and nasal sides of the optic disc, especially macular region and area superior to the macular. The CT was reduced in the large triangular region inferior to the optic disc (Fig. 2). Meanwhile, the CVI in the choroidal thickened region was significantly increased (Fig. 2 bottom), indicating that Sattler’s layer and Haller’s layer are the main reasons for the increased CT. Sumit Randhir Singh et al. demonstrated a positive correlation between CVI and SFCT^22^, similar to our results. Choroid thinning inferior to the optic nerve head with a large triangular shape may be induced by the downward closure of the fetal chorioretinal fissure occurring at 16 weeks^14^ and related to the nature of the choroidal vasculature and the presence of a choroidal watershed zone^23^.

Currently, some researchers proposed that the normal and thickened pathological choroidal limit was 395 μm below the fovea as the threshold^24^. Sara Touhami used widefield OCTA to measure the CT of healthy people under 55 years of age with a range of 9 mm × 9 mm and discovered that 30% SFCT was larger than 395 μm^16^. Eleven eyes (5.2%) of our volunteers’ SFCT were larger than 395 μm, and these subjects were aged 20–50. B-scan images of these subjects did not reveal any pathological changes correlated with pachychoroid, such as pathological dilatation of Haller's layer, thinning of Sattler's layer and capillary layer^25^, and any pathological changes in the RPE and outer retina (Fig. 4). Sara Touhami et al. uncovered that TCP mostly appeared in the superior and temporal regions outside the fovea (72.2%). Our study was slightly inconsistent. Specifically, more than 3/4 of TCP of the subjects had located above the fovea owing to our larger observation range that most of them were beyond the 9 mm × 9 mm range. Our results can more accurately reveal the positional relationship between the TCP and the fovea, which is of great significance for us in analyzing CT more comprehensively and accurately, especially in the research on the pachychoroid disease spectrum.

Second, an increasing trend of MCT was observed in the younger population under 30 years, similar to the outcome of Scott A. Read et al.^26^. This was associated with the changes in retinal structure and physiological demands^6,27^. We hypothesize that the temporary increase in MCT in the 20–29 years age group is due to the fact that the choroid is in a continuous developmental phase since infancy until puberty, when development gradually stabilizes and thus MCT reaches a peak value which is 20–29 years of age. After puberty, growth ceases in normal subjects, and early choroidal thinning occurs due to the reduced metabolic demand of the retina with age. However, the explanation mechanism of early choroidal thinning is still inconclusive, and we will expand the sample size for further study in the next step. The changes in choroidal thickness between the ages of 30 and 50 did not present a statistical correlation with age, and the MCT was significantly negatively correlated with age in subjects over the age of 50, similar to the conclusion of Jiamin Xie et al.^28^.

Due to the limitation of observation scope, few studies compared the decline of MCT with age in the macular region with other regions. In 2018, Wenjia Cai et al. conducted a 2-year longitudinal study on healthy volunteers over 30 years old^29^. They discovered that the greatest 2-year change in MCT was in the group aged 50–59 within a 6 mm × 6 mm area centered on the macula. Nevertheless, they did not compare it with the larger surrounding area. In our study, the MCT of the group aged 20–29 was used as the baseline, and the difference between the MCT in each region of the group aged ≥ 50 and the baseline were calculated. The left and right eye results separately was due to our consideration of potential asymmetry in the interocular CT within different areas^21^. The LSD test results (Fig. 6) revealed that the decrease in MCT in the macular region was greater than in others, for both the left and right eyes. The decrease in CT generally indicated a reduction in choroidal blood density and blood supply, and the loss of choroidal vasculature weakened the ability of the choroid to supply oxygen and metabolites to the RPE and retina^30^. The high metabolic demand for nutrient supply to the macula requires rapid and intensive filling of choroidal capillaries and choroidal arteries^29,31^. In summary, it is hypothesized in our study that the accelerated loss of choroidal vasculature in people aged > 50 leads to a decrease in the ability of the choroid to supply oxygen and metabolites to the RPE and retina^30^. Then, the decrease in MCT was more pronounced in the area of the macula with a high metabolism. This condition may also be closely related to the development of AMD^32^, which is a crucial reason for blindness in adults aged > 50^33^. AMD in the early period mainly manifests as loss of choroidal vascular endothelial cells. Investigators compared the eyes with reticular pseudo drusen (RPD) to that of normal eyes, suggesting that fibrotic replacement and horoidal vascular depletion may play a vital role in the pathogenesis and progression of disease^32^. Although choroidal thickness decreases after the age of 50, declines significantly faster in the macula compared to other areas, providing a new explanation why the majority of choroidal neovascularization in AMD occurs in the macula. Presbyopia is another common physiological phenomenon associated with age, mostly in people over the age of 50. Uncorrected presbyopia may relate to hyperopic retinal defocus for near objects. With prolonged periods in a near visual, it will possibly reduce choroidal blood flow, and thus causes the reduction of CT. Whether retinal hyperopic defocus has a greater effect on the choroidal structure of the macula will be the focus of our further longitudinal studies.

This study has the following shortcomings. The sample size is small, especially in the male population. Bafiq R et al. reported that the choroidal thickness declined with age but did not show any sex based differences^34^. From this, we believe that sex differences do not contribute to the finding that choroidal thickness varies with age. In our experiment, data were collected between 10–12 am and 2–5 pm, and the effect of circadian rhythms was neglected^35^. However, the number of people in the morning and afternoon was roughly close, so the time rhythm minimized the effect on the results of our experimental study. These factors may impact the results and will be further controlled in future studies. The limitations of this cross-sectional observational study make it difficult to take a more in-depth exploration of some interesting findings, such as the age of peak choroidal thickness development, the relationship between choroidal thickening and thick chorioretinopathy^36^, and the role of greater thinning of the macula relative to other parts in the early onset of AMD development. Thus, the sample size will be expanded for long-term longitudinal follow-up on certain meaningful issues.

## Conclusion

The results of our study demonstrated that the choroid is thickest in the macular region and supratemporal region, followed by the nasal side of the optic disc and the area below the optic disc. MCT decreased more rapidly in the macular region compared to other regions after age 50 years old. The 120° UWF SS-OCTA can be used to observe the distribution of choroidal thickness in the range of 24 mm × 20 mm and its variation with age, providing a reference for choroidal and retina-related diseases.

## Supplementary Information

Supplementary Information.

## Data Availability

The datasets used and/or analyzed during the current study available from the corresponding author on reasonable request.
